# Stimulus Level during Endurance Training: Effects on Lactate Kinetics in Untrained Men

**DOI:** 10.1155/2018/3158949

**Published:** 2018-12-02

**Authors:** Michael Tuttor, Simon von Stengel, Michael Hettchen, Wolfgang Kemmler

**Affiliations:** ^1^German Rugby-Union, Deutscher Rugby-Verband e.V. (DRV), Ferdinand-Wilhelm-Fricke-Weg 10, 30169 Hannover Rugby-Verband, Germany; ^2^Institute of Medical Physics, Friedrich-Alexander University Erlangen-Nürnberg, Erlangen, Henkestrasse 91, 91052 Erlangen, Germany

## Abstract

**Background/Objective:**

Not only but particularly due to their time efficiency, High-Intensity Interval Training (HIIT) is becoming increasingly popular in fitness-oriented endurance sports. The purpose of this study was to determine the effect of a HIIT running program versus a Moderate Intensity Continuous Exercise (MICE) training running program (16 weeks each) on lactate kinetics in untrained males.

**Methods:**

65 healthy but untrained males (30-50 years, BMI: 27.2 ± 3.7kg/m^2^) were randomly assigned to either an HIIT (n=33) or a waiting-control/MICE group (n=32). HIIT consisted of intervals and intense continuous running bouts at or above the individual anaerobic threshold (IANS, 95-110% of IANS-HR), while MICE focused on continuous running at 70-82.5% IANS-HR. Both programs were adjusted for “total workload”. Study endpoints were time to IANS and time from IANS till “time to exhaustion” (TTE) as assessed by stepwise treadmill test.

**Results:**

In both exercise groups time to reach IANS (MICE: 320 ± 160 s versus HIIT: 198 ± 118 s) increased significantly (p<.001), with the groups differing significantly (p<.001). Time from IANS until TTE was prolonged significantly among the HIIT group (27 ± 66s, p=.030), while among the MICE group a significant reduction of time from IANS until TTE (59 ± 109s; p=.017) was determined. Between-group difference is significant (p=.003) for this parameter. In both groups TTE increased significantly (HIIT: 27.2 ± 17.7% versus MICE: 29.0 ± 19.4%, both p<.001) at a similar level (p=.279).

**Conclusion:**

HIIT and MICE protocols, when adjusted for total workload, similarly increased running performance in untrained male subjects; however, the underlying mechanisms differ fundamentally. Due to its effects on aerobic and anaerobic performance improvement, HIIT can be recommended for untrained individuals as a time-efficient alternative or complementary training method to MICE. However, our protocol did not confirm the general superiority of HIIT versus MICE on the key endurance parameter “time to exhaustion” that has been reported by other comparative exercise studies.

## 1. Background

High-Intensity Interval Training (HIIT) methods are becoming increasingly popular in fitness-oriented endurance sports, not least because of their time effectiveness and interesting nature [1, 2]. The method is basically characterized by intermittent loading phases of high to maximum stimulus intensity that alternate with “recovery phases” of lower, but not necessarily low, intensity.

Studies with different groups have shown that HIIT has significant and in some cases significantly more favorable effects on maximum oxygen consumption ($\dot{V}$O_2_max) than the moderate-intensive continuous exercise (“moderate intensity continuous exercise”: MICE) [3–7].

The present RUSH Study (Running Strengthens the Heart) [8] also confirmed the significant superiority of HIIT on this aspect of endurance performance in untrained middle-aged men. However, HIIT and MICE showed themselves to be comparable in terms of endurance performance, measured by treadmill level test as “time to exhaustion”.

In addition to $\dot{V}$O_2_max, anaerobic capacity (ANC) and running economy (RE) also make an important contribution to endurance performance [9]. In fact, as with Iaia et al. [10], but unlike other similar studies [5, 11–13], the RUSH Study showed a significantly more favorable development of RE in MICE compared with the HIIT group. Corresponding effects on lactate kinetics as a central aspect of anaerobic capacity were not observed in the present study, but a positive influence of HIIT on ANC, especially for HIIT formats with high/maximum stimulation and a load duration of between 20 seconds and 5 minutes/interval, was ascertained [3, 9, 14, 15]. At this point at the latest, it should be noted that several intensity-oriented concepts are subsumed under the term HIIT, which may lead to significantly different adaptational reactions. Buchheit and Laursen distinguish between several HIIT formats [16] to which they assign different metabolic relevance. In addition to the classic HIIT of endurance-oriented sports [17], new HIIT concepts have been established under the terms “Repeated Sprint Interval Training” (RST) and “Sprint Interval Training” (SIT), which are mainly used in ball sports [18, 19]. Without going into the different formats, HIIT is therefore to be seen as a relatively heterogeneous interval training method, the interval duration of which varies between approx. 5 seconds and 8 minutes [15, 20]. The applicability of HIIT to the untrained is not uncontroversial [21, 22].

Within the RUSH Study, considering the status of our target group (untrained, healthy men 30-50 years; predominantly overweight), the load type (running), and the training objective (10 km running competition), we regarded the execution of a complex HIIT protocol including SIT and RST components as possibly too demanding, so we limited ourselves mainly to interval training forms in the range of ≥90 sec.

Following the spiroergometric estimation of the performance-determining endurance components “maximum oxygen uptake” and “work economy” [8], it is the aim of this paper to evaluate the effects of HIIT versus MICE on aerobic and anaerobic “efficiency” by means of lactate kinetics. The “anaerobic threshold” (IANS), which is determined via the “lactate performance test", provides insights into the metabolism [23–25]. Nevertheless, after exceeding the IANS, physical activity is not accompanied by purely anaerobic energy supply (product of the capacity of the cardiorespiratory system to supply oxygen and the capacity of the skeletal muscles to utilize oxygen [26]) (maximal amount of ATP resynthesis via anaerobic metabolism during a short duration maximal exercise [27]) in the true sense of the word [25, 28]. In this article we equate the aerobic efficiency (AE) with the performance range up to reaching IANS, the anaerobic efficiency (ANE) with the performance range after reaching IANS up to the end of performance (TTE). Following this premise, we investigated the extent to which a moderate HIIT (in terms of stimulus intensity) compared with MICE leads to a “shift" of the IANS and thus to training-induced metabolic adjustments. In this context, we are not aware of any other study, which has investigated this question in a comparable design with an untrained adult population and a sufficient number of test persons.

On the basis of comparable changes in “duration up to the end of performance” in the present study [8], our hypothesis was that (1) MICE leads to a higher increase in aerobic performance, defined as time under load (TUL) until the anaerobic threshold is reached, while (2) HIIT leads to a stronger increase in anaerobic performance, defined as time (TUL) between reaching the anaerobic threshold and aborting the physical activity (TTE).

## 2. Material and Methods

The Running Strengthens the Heart (RUSH) study was a controlled intervention study using a randomized two-group design. We evaluated the effects of two 16-week running training phases of varying intensity on endurance performance as well as on spirometric, metabolic, and cardiac parameters. However, we also implemented a waiting-control group in order to estimate the overall effect of the exercise methods. Nevertheless, in this article we specifically focus on the effects of a HIIT versus a MICE protocol on lactate kinetics in the stress test. The study was conducted at the Institute of Medical Physics of the Friedrich-Alexander Universität Erlangen-Nürnberg (FAU). The study protocol was approved by the Ethics Committee of the FAU (application 4463); all study participants gave their written consent. The study is registered at www.clinicaltrials.gov (NCT01406730).

### 2.1. Study Endpoints

#### 2.1.1. Primary Endpoint

- Change of time under load (TUL) between reaching the anaerobic threshold and the point at which the load is aborted (TTE).

#### 2.1.2. Secondary Endpoints

- Change in the duration of time under load (TUL) until the anaerobic threshold (IANS) is reached.
- Change in the IANS speed.
- Change in maximum lactate concentration at the end of the load.

### 2.2. Participants

**Figure 1:**
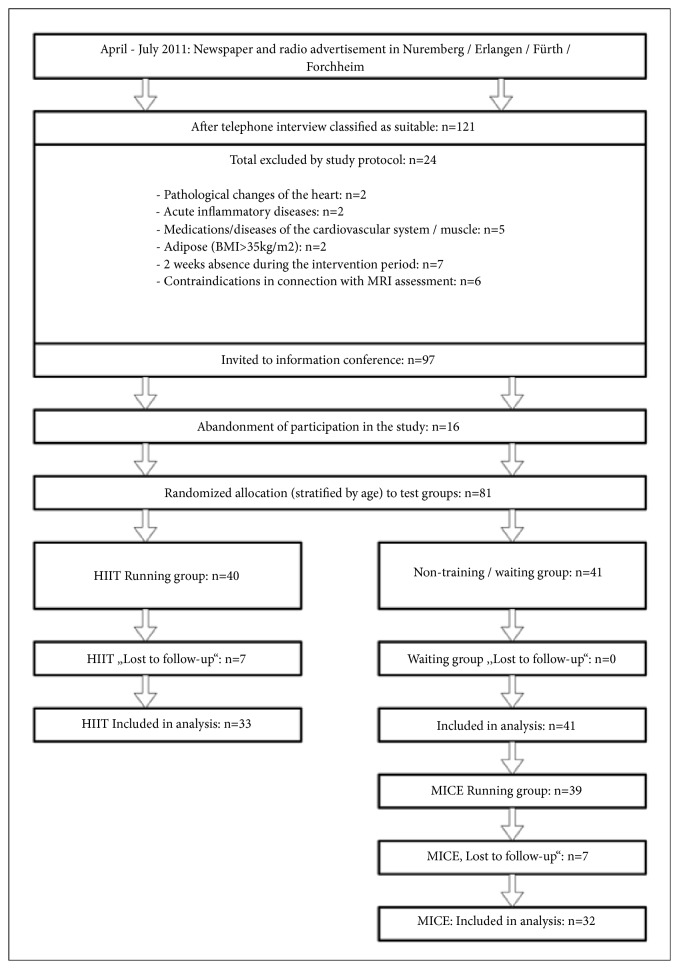
Flowchart of the “RUSH Study”.

Figure 1 gives the participant flow of the study. Men aged 30-50 years living in the area of Erlangen-Nürnberg (Germany) were contacted by personal mail. One hundred and twenty-one males responded and were assessed for eligibility. Following our main inclusion criteria of (a) male, (b) 30-50 years old, (c) untrained status for longer than 2 years (i.e., ≤1 endurance exercise sessions; ≤2 exercise sessions/week in total), and the corresponding exclusion criteria of (a) inflammatory diseases, (b) pathological changes of the heart, (c) medication/diseases affecting cardiovascular system and/or muscle, (d) severe obesity (Body Mass Index > 35 kg/m^2^), (e) very low physical capacity (<100 Watt/3 min at bicycle ergometry), (f) more than 2 weeks of absence during the interventional period, 97 men were invited to a meeting with presentation of our study protocol. Sixteen subjects were unwilling to join the randomization procedure and quit the study before randomization. Thus, 81 subjects were randomly assigned (computerized block randomization stratified for age) to two subgroups: (a) high-intensity (interval) training (HIIT) group and (b) waiting-control group/moderate intensity continuous exercise (MICE) group (Figure 1).

Table 1 gives the baseline characteristics of the participants. Of importance, variables that may have impacted our results did not vary between the groups.

### 2.3. Measurement

All examinations were carried out by the same investigator, in identical sequence, on the same premises, at the same time (±1 h), with the same test device (see below). The examiners were also forbidden to inquire about the status (HIIT or waiting group/MICE) of the test persons.

#### 2.3.1. Anthropometric Data

Height (Holtain Ltd., Crymych, GB) and perimeter values (Prym, Stollberg Germany) of the test persons were measured with calibrated instruments. Body weight (in kg), total and regional fat-free body mass (in kg), and body fat (in %) were measured by means of modern multifrequency bioimpedance measurement (Biospace Inbody-230, Seoul, Korea). Intra Class Correlation (ICC, test-retest) of total fat-free body mass as assessed by the Inbody-230 was 0.88 for a comparable cohort of men 30-50 years old; ICC for percent body fat was comparably high (0.86).

#### 2.3.2. Endurance Capacity and Lactate Diagnostics

The endurance capacity was measured by treadmill level test (Technogym, Gambetolla, Italy) up to the subjective load. As objective loading criteria, all tests carried out showed a maximum respiratory quotient of >1.05 and an O_2_ breath equivalent of >30.

The initial load was selected depending on the individual performance capability and was consistent (base and control measurement) at 7 or 8 km/h for the individual test person to ensure a load duration of at least 9 minutes. The treadmill incline was 1%, every 3 minutes the speed was increased by 1 km/h.

Heart rate monitors (Polar RS-400, Kempele, Finland) were used to continuously record the heart rate. The heart rate of the last 30 s of each exercise level was included in the data analysis.

At the end of each exercise stage, the blood lactate concentration was determined. The blood collection at the earlobe was carried out via 20 *μ*L end-to-end plastic capillaries, which was then expelled into 1 mL reaction vessels for analysis. The diagnostic device used is based on enzymatic-amperometric chip-sensor technology (Biosen-5130, Gemar, Celle, Germany).

#### 2.3.3. Lactate Threshold Determination

The individual anaerobic threshold (IANS) was determined as minimum lactate + 2.0 mmol/l as our research subject as well for generating a reference value within the framework of training planning. It is quite similar to the model of Dickhuth et al. [29]. Speed, heart rate (HR), and lactate values at this threshold were calculated using the software “Threshold” (Wassermann, Bayreuth, Germany).

#### 2.3.4. Spirometry

Open spirometry (Viasys, Conshohocken, USA) was used to continuously determine the $\dot{V}$O_2_ with a breath-by-breath procedure. The data of the last 30 s of each load level were included in the data analysis. Running economy (RE) was assessed before and after intervention for the last 30 sec of the second stage of the test (5:30 to 6:00 min) at identical running speed, which was consistently kept below the anaerobic threshold. Running Economy was calculated using VO_2_*∗* kg^−0.75^*∗* min^−1^.

VO2max was considered as a plateau of oxygen consumption, defined as any 30 s period that was no more higher than the preceding 30 s period.

#### 2.3.5. Questionnaire/Influencing Factors/Nutrition

Among other things, sociodemographic factors, dietary habits, illnesses, health risk factors, sports activities, and physical activity were recorded via a validated questionnaire [30]. A final questionnaire captured changes that could have influenced our study endpoints (e.g. taking medication and sports activities).

At the beginning and end of the study, the participants' nutrition was recorded and evaluated via a 4-day nutrition protocol (Nutri-Science, Hausach, Germany).

**Figure 2:**
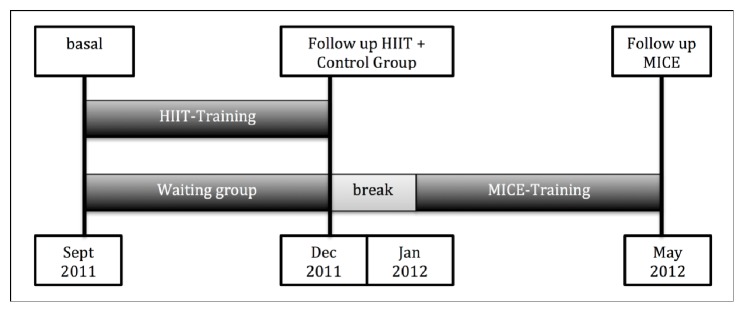
Study design of the RUSH Study.

Figure 2 shows the design of the intervention. Both training groups trained 16 weeks based on individualized training protocols. These training plans, which included intensity, extent, density, and frequency of stimulus, were analyzed every 4 weeks to monitor the quantity and quality of the training. The stimulation level was specified by the HR. For this purpose, the participants were provided with a heart rate monitor (Polar RS 400, Kempele, Finland). The participants' heart rate monitors were randomly sampled (each training session (TS) 15-20 datasets) to assess compliance with the training guidelines. The training volume was gradually increased in both training groups by extending the time under load per training unit (TS) (from initial 30-35 min up to 90 min/TS) and the number of weekly training units. During the first four weeks, 2 TS/week and during the second four-week period 2 to 3 TS/week were completed. After this initial phase with progressively increasing volume, a nonlinear training protocol with 3-4 TS/week was implemented during the last 8 weeks of the intervention period. Participants were required to participate in a supervised TS at least once a week and were free to participate in further joint TS. In total, a maximum of 49 TS could have been completed during the intervention period. The intensity of the stimulus was determined individually according to the threshold concept described above by means of a training plan. In order to measure the isolated influence of the stimulus intensity on our endpoints, the duration of the training protocols (HIIT versus MICE) was adjusted such that the physical work became comparable (isocaloric).

### 2.4. Intervention: Training Groups

#### 2.4.1. High-Intensity Interval Training (HIIT)

The stimulus intensity of the HIIT training varied mainly in the range of 95-110% IANS-HR. In addition to the interval method with an interval duration of 90 s-12 min and a pause duration of 1-3 min (70-75% IANS-HR), continuous loads (25-45 min) were also used in the IANS zone (threshold runs). In total, the training components were distributed as follows:

1. Loads above the IANS (interval >102.5% of the IANS-HR): approx. 40% of the total volume.
2. Loads in the range of the IANS (interval/continuous load 95-102.5% of the IANS-HR): approx. 35% of the total volume.
3. Low-intensive loads clearly below the IANS (70-75% of the IANS-HR): approx. 25% of the total volume.

The training components of the “low-intensive” exercise zone consisted exclusively of the load contents “warm-up/running” and “active break” as well as (in total two) regenerative training units.

#### 2.4.2. Moderate Intensity Continuous Exercise (MICE)

Although the intensity of training varied somewhat more markedly (≈ 70-100% IANS-HR) than in the HIIT group over the intervention period as a whole, the training methodological emphasis was in the range of 70-82.5% of IANS-HR. Training mainly entailed an extensive endurance element, i.e., extensive “Fartlek” (35-90 min) and only sporadic loads in the area of the IANS (especially to verify it, see above). Overall, the training components were distributed as follows:

1. Loads above the IANS (>102.5% of the IANS-HR): 0% of the total volume
2. Loads in the area of the IANS (95-102.5% of the IANS-HR): 5% of the total volume
3. Loads below the IANS (82.5-95% IANS-HR): 10% of the total volume
4. Loads in the low-intensity range (70-82.5% IANS-HR): 85% total volume

Table 1 shows the base characteristics of the two training groups. No significant intergroup differences were recorded for the given parameters.

### 2.5. Statistical Procedures

The present study has a randomized two-part design. The calculation of the formal number of cases of the study is based on what is probably the “most critical” endpoint of the study, the end diastolic volume of Heart-MRI measurement, a quantity that is not further addressed in this paper. The calculated number of cases of 40 persons per group generates a statistical power (*α*: 5%) of 96%, based on the expected intergroup difference of 10 ± 12% for the questions addressed. Furthermore, the hypotheses cited above attribute this contribution primarily to the effect of stimulus intensity, so that the control group data are no longer taken into account here. A “Completer Analysis” was carried out, in which all persons (independently of their “compliance”) who participated in the control (i.e., 16 week follow-up) assessment were taken into account.

After checking the normal distribution assumption by means of graphical (boxplot, histogram) and statistical tests (Shapiro Wilks-Test) and, if necessary, corresponding log transformations of nonnormally distributed variables, changes within the groups were analyzed by means of a T-test for dependent samples. Intergroup differences between HIIT and MICE were calculated by independent (Welch) T-Tests, based on absolute differences within the groups between baseline and 16-week follow-up adjusted for baseline data. A significance level of 5% was set. All data were analyzed with SPSS version 22.0 (SPSS INC, Chicago, USA).

## 3. Results

Thirty-three (33) participants of the HIIT (83%) and 32 participants of the MICE group (80%) were included in the final analysis. Nine persons discontinued the study because of injuries or orthopaedic problems (HIIT: n=4 versus MICE: n=5). In eight cases, this was related to running training. Three persons (HIIT: n=2 versus MICE: n=1) fell ill and were no longer available for follow-up measurements. One subject in each of the training groups discontinued training for undisclosed reasons. Two subjects of the control/waiting group lost interest in further study participation even before the start of the MICE training period.

### 3.1. Training Characteristics

Table 2 shows the training frequency, training volume and work done over the intervention period of both training groups. Due to the higher stimulus intensity of the HIIT group, the physical “work” was comparable, despite different training duration and same training frequency.

Both training groups carried out the training sessions in accordance with the protocol as far as possible. The attendance rate for the HIIT group was 83 ± 11% (40.5 ± 5.4 TS; 27 to 49 TS); the corresponding rate for the MICE group was 82 ± 12% (40.3 ± 5.9 TS 25 to 48 TS) (Table 2). The 4-week training sessions also showed no significant longitudinal changes or differences between the groups with regard to the attendance rate. It was noticeable that the HIIT group preferred not to carry out the few intensive continuous power units (n=9) in the anaerobic threshold range with the planned training frequency (56% execution rate), while in the MICE group there was no tendency to favor the implementation of any particular training content.

## 4. Study Outcome

In both groups, the duration until load abort increased comparable (p=. 297) and significantly (p<.001) by 226 ± 120 s (MICE) and 261 ± 143 s, respectively.

The significant (p<. 001) increase in the time to reach IANS (TUL/IANS) in both training groups was more pronounced in the MICE group with an increase of 320 ± 140 seconds (p<.001) than in the HIIT group: 198 ± 128 seconds. In the HIIT group, the time from reaching IANS until load abort (IANS/TTE) increased significantly (p=.030) by 27 ± 66 s, whereas in the MICE group a significant (p=.011) reduction of 59 ± 109 s was observed. The difference between the subgroups was significant (p=.003).

Our hypothesis (1) can thus be confirmed. Our assessment of hypothesis (2) regarding the exclusive effects of HIIT on an increase in ANE is also verified by the study results (Table 3).

Speed at the IANS increased significantly in both groups. However, the improvement was significantly higher in the MICE group than in the HIIT group. A slight, nonsignificant increase in the maximum lactate concentration was recorded for both training groups. No differences between the groups were recorded with tending higher lactate values in the HIIT group (Table 4).

Not addressed in this article, however potentially important in the interpretation of the results, running economy (RE) nonsignificantly increased (3.0 ± 10.3%, p=.148) in the HIIT group and significantly decline (-5.7 ± 9.3%, p=.005) in the MICE group. Differences between the two groups were significant (p=.003).

### 4.1. Confounding Variables

Weight and skeletal muscle mass changed relevantly with significant differences between the training groups (weight: HIIT: −1.3 ± 2.5% versus MICE: −2.8 ± 2.7%, p=.028) (skeletal muscle mass: HIIT: +0.5 ± 2.3% versus MICE: −1.3 ± 2.0%; p=.002). No significant changes in lifestyle, diet, physical activity, or exercise were recorded during the intervention period. The outcome effects are therefore primarily attributable to the intervention.

## 5. Discussion

The intention of this article was to investigate a relatively moderate HIIT format [16], which is suitable for novice runners, with regard to the training effect on aerobic and anaerobic performance as compared with MICE training. For better comparability, both training methods were “adjusted” with respect to the workload, an approach that led to relatively long HIIT intervals despite high intensity. In summary, both training methods led to a comparable significant increase in endurance performance (TUL: HIIT: 27.2 ± 17.7% versus MICE: 29.0 ± 19.4%), defined as time to load abortion but by significantly different paths. Although HIIT and MICE show significant improvements in aerobic performance (AE) defined as “time to reach IANS” (Table 3) or a significant “shift to the right” of IANS (Table 4), both values show significantly better results for the MICE group. This finding, that a HIIT is able to have a significant but less favorable effect on AE than MICE, is at odds with the findings of several studies [9, 14, 31]. In contrast, (only) HIIT induces an increase of the anaerobic performance (ANE), defined as TUL between reaching the IANS and end of load. In essence, this corresponds to the improvement in AE and ANE demonstrated by Tabata et al. [6] and others [9, 14, 15, 32] by a HIIT. Since spiroergometry data or other parameters on the topic are usually available (see above), however, the classification of our results should be viewed with care. The HIIT stress protocol and the patient samples of the aforementioned (HIIT) interventions differ from the protocol presented here, so that a cautious interpretation seems all the more appropriate. Nevertheless, the available knowledge is favorable.

That HIIT causes an increase in the performance of fixed lactate values among trained persons [5, 33] or at the “LT threshold” among athletes [34] mainly supports our result of HIIT raising the IANS in the case of untrained persons.

In general, however, the interpretation of workout-induced lactate threshold modifications is always difficult and we consider it plausible that stimulation level and extent of a workout have different effects on the threshold of untrained persons compared with athletes [15].

Moreover, the lack of standardization in threshold determination is always critical. The large number of existing threshold concepts, inconsistent “stress protocols", their inadequate description, and the nonexistent terminology standards in the field of medicine and sports science do not permit a clear evaluation of the study situation and thus make it difficult to classify the study results [23, 35].

Considering the change in the maximum lactate concentration at the time of load abort, the HIIT group shows a partial increase (p=.067) with no significant difference to the MICE group, where there is no significant change in this value. The data of the available literature are rather inconsistent in this respect: while Sperlich et al. [36] and Stoggl et al. [37] reported significant HIT-induced lactate changes, Helgerud et al. [5] reported no significant changes after two different HIIT formats. The same applies to MICE protocols for which Sperlich et al. [36] reported a reduction after moderate-intensive high-volume training with 9-11-year-old performance swimmers.

As a representative parameter of cardiorespiratory fitness, $\dot{V}$O_2_max is the usually applied performance parameter in the research field [5, 38–40]. There is no question that endurance capacity manifests itself in $\dot{V}$O_2_max. However, it arises not only from the $\dot{V}$O_2_max, but also from the determinants discussed here, which come to light with a view to the submaximal load situation (see above). Last but not least, despite the comparable increase in TUL, significantly higher $\dot{V}$O_2_max absolute values were achieved through HIIT compared with MICE [8]. Based on our results for the optimization of RE by MICE [8], we assumed that the observed effects are also represented in lactate kinetics. The available results go some way to confirming this. The spirometric variation of the RE is also revealed to some extent in the correspondingly modified lactate threshold. With an increase of 320 ± 160 seconds (versus HIIT: 198 ± 128 seconds), MICE has a more significant (p<.001) effect on the IANS (as a presumed expression of economic metabolic processes in the submaximum load range) than HIIT. Nevertheless, the spirometry data of the present study also show a significant decrease in RE by HIIT although the training method also achieved an increase in AE to a highly significant extent. An increase in lactate-determined AE obviously does not necessarily mean an improved RE. Here, the term “economisation effect” must also be considered in a differentiated way. Conversely, an economisation of the submaximal metabolic process does not have to be accompanied by an increase in IANS. It is also conceivable that within the measuring range of the RE determined spirometrically (the RE was determined on the basis of the following calculation: $\dot{V}$O_2_ (ml x kg – 0.75 x min^−1^) [41] of the last 30 s of the second load stage (5:30 to 6:00 min)) an improved ANE leads to the load process being unable to proceed so economically, since the metabolism is trained to maximize performance per se. Accordingly, we conclude that the main adjustment takes place in the respective main load areas. As our measurements also show, both types of training can improve the athletic performance of the untrained, determined by the TUL, comparably effectively (p=.297). With regard to their effectiveness in improving the physical performance of untrained subjects, the results are in line with the current state of research [9, 32, 42].

Targeted measures must always be based on a target-group-oriented weighting of all the variables. In this sense, our training protocol was less intensive and should also be able to be carried out independently in the field.

A HIIT protocol can be described with up to nine variables: the exercise as such, the respective intensity and duration of the work and rest interval, the number of repetitions and series, and the duration and intensity of the series breaks [16, 19].

We were convinced that untrained sports beginners should avoid extremely high intensities, as this would also reduce susceptibility to injury at lower levels of stress (cf. special features and limitations-point (d)). This topic was also addressed by Lunt et al. [43]. As the authors show, success and implementation of the HIIT study training protocols are governed by different rules “in real life”. In overweight adults, only a very small improvement of the $\dot{V}$O_2_max could be achieved. Adherence was low and minor training injuries occurred. Foster et al. [32] observe a decrease in the “joy” of HIIT among the untrained subjects over an 8-week intervention period. However, to conclude that there is an acceptance problem with the HIIT method in the case of untrained persons would disregard further current research results, because it has also been shown that untrained persons prefer HIIT as a training variant [1, 44].

The main strengths of our study are (a) unlike many other studies [7, 11, 13, 32, 45] it offers a sufficiently high sample size and long duration of intervention, which makes it possible to identify clinically relevant differences, (b) regular monitoring and adjustment of the load specifications via HR, (c) a training protocol that can be easily and safely incorporated in the training routine, (d) the controlled randomized design with waiting/control group, (e) the recording of relevant parameters of endurance capacity and its determinants, and (f) the consistent recording of covariates (medication, diseases, nutritional behavior, lifestyle, and training).

Particular features and limitations of our study are as follows: (a) The lack of a specific recording of the ANE, e.g., via “Wingate-Anaerobic-Test” as well as the fine adjustment of the IANS via maxLass/MLSS-Test. This could not be achieved for economic reasons. (b) In addition, the determination of ventilatory thresholds would contribute to the additional verification of the available results and would provide further plausibility. Thus, the working group “cardiopulmonary exercise testing” also calls for a combination of both diagnostic methods in the sense of better and more precise diagnostics [25]. (c) The delayed execution of the training protocols (Figure 2). Here it is possible that seasonal changes in physical activity may affect our results. However, the data for this parameter at base and at the end of the study do not indicate any change. (d) The relatively high drop-out rate (20%) due to training-induced injuries and discomfort (comparable in HIIT and MICE). (e) The only random checking of the given stimulation level by reading out the heart rate monitors. In this respect, it is possible that the implementation of the stimulus level was not always in accordance with the protocol. (f) From a purely statistical point of view, applying the intention-to-treat principle would be preferable to our “Completer” approach. Furthermore, the formal case number calculation was carried out with a (cardiac) parameter which was not the subject of the present study.

## 6. Conclusion

In summary, it can be concluded that an increase in the AE of metabolism can be induced by both MICE and HIIT. Metabolic processes are optimized mainly in the most frequently trained area, i.e., in the main requirement area. High training stimuli can contribute to performance improvement in a time-efficient way and address different physiological adaptations depending on the level of stimulation. To our knowledge, HIIT can optimally represent the complementary component to MICE or HIIT regimes and, depending on the objective, it can contribute to the efficient optimization of performance. Depending on the intention of the athlete, an easy-to-implement, extended MICE protocol could also be the preferred training option in terms of increasing energy consumption. Considering that MICE exceeds our HIIT program by only 5.5 minutes in terms of average training time/TS, this is a conceivable approach. Another desirable effect was the best possible transfer of our intervention measures into everyday training and the increase in compliance beyond the end of the intervention. Researching this in a follow-up study would be beneficial and worthwhile in terms of a more comprehensive understanding of the potential long-term effects of high-intensity training methods.

## Figures and Tables

**Table 1:** Baseline characteristics of subject groups. ^1^However, in five subjects each per group a plateau of oxygen consumption could not be determined.

**Characteristics**	**HIIT (n=33)**	**MICE (n=32)**
Age (Y)	43.9 ± 5.0	42.9 ± 5.1
Height (cm)	181.1 ± 7.0	181.6 ± 5.3
Weight (kg)	91.5 ± 14.0	89.5 ± 12.3
Body fat (%)	25.4 ± 5.7	24.7 ± 6.2
Lean body mass (kg)	68.2 ± 8.0	68.6 ± 7.3
Energy Intake (kcal/d)	2595±738	2737±592
Activity (Index)	2.8 ± 1.3	2.7 ± 1.2
Activity (min/week)	32.2 ± 31.9	33.0 ± 36.3
Rel. $\dot{V}$O_2_max (ml/kg/min)^1^	36.9 ± 5.6	39.3 ± 5.5

**Table 2:** Training characteristics HIIT versus MICE.

**Characteristics**	**HIIT**	**MICE**	**P**
Frequency (TS/16 weeks)	40.5 ± 5.4	40.3 ± 5.9	.926
Volume (min/16 weeks)	2092 ± 298	2303 ± 352	.012
Work (kcal/16 weeks)	28966 ± 5228	30479 ± 6566	.317

**Table 3:** Results for the study endpoints. Mean values, standard deviations, and mean difference between the groups with 95% confidence interval, as well as significance values of the intergroup comparison and corresponding effect size (ES). Numerical value in parentheses: Significance value of the change within the groups between base and final (16-week measurement).

	**HIIT**	**MICE**	**Absolute difference MV (95**%**-CI)**	**p**	**ES** **d**′
	**MV ± SD**	**MV±SD**			
**Time to reach IANS [s]**					

Baseline	482 ± 235	511 ± 264	-----	-----	-----
16 weeks	680 ± 228	831 ± 268	-----	-----	-----
Difference	198 ± 128 (p=.001)	320 ± 140 (p=.001)	122 (51 to 192)	0.001	0.91

**Time from attainment of the IANS until load abort [s]**					

Baseline	351 ± 144	392 ± 152	-----	-----	-----
16 weeks	378 ± 147	333 ± 134	-----	-----	-----
Difference	27 ± 66 (p=.030)	-59 ± 109 (p=.011)	86 (34 to 136)	0.003	0.95

**Table 4:** Results for the secondary endpoints “velocity at anaerobic threshold” and “maximum lactate”. Mean values, standard deviations, and mean difference between the groups with 95% confidence interval, as well as significance values of the intergroup comparison and corresponding effect size (ES). Numerical value in parentheses: significance value of the change within the groups between base and final (16-week measurement).

	**HIIT**	**MICE**	**Absolute difference MV (95**%**-CI)**	**p**	**ES**
	**MV ± SD**	**MV ± SD**			**d**′
Speed at IANS [km/h]					

Baseline	9.68 ± 1.24	9.72 ± 1.47	-----	-----	-----
16 weeks	10.77 ± 1.33	11.50 ± 1.44	-----	-----	-----
Difference	1.10 ± 0.65 (p=.001)	1.78 ± 0.83 (p=.001)	0.69 (0.32 to 1.07)	.001	.91

Maximum lactate (mmol/l)					

Baseline	7.91 ± 2.22	7.64 ± 1.75	-----	-----	-----
16 weeks	8.33 ± 2.08	7.72 ± 1.47	-----	-----	-----
Difference	0.42 ± 1.27 (p=.067)	0.08 ± 0.90 (p=.616)	0.34 (-.21 to .89)	.222	.31

## Data Availability

The anonymized data used to support the findings of this study are available from the corresponding author upon request.

## Abbreviations

AE: Aerobic efficiency
ANE: Anaerobic efficiency
ANC: Anaerobic capacity
Cm: Centimeter
HIIT: High-Intensity Interval Training
HR: Heart rate
IANS: Anaerobic threshold
Kg: Kilogram
MICE: Moderate intensity continuous exercise
Min: Minute
mmol/l: Millimole per liter
RE: Running economy
RST: Repeated Sprint Interval Training
S: Second
SIT: Sprint Interval Training
TS: Training session
TTE: Time to exhaustion
TUL: Time under load
$\dot{V}$O2max: Maximum oxygen consumption.
